# The complement system: an evolution in progress

**DOI:** 10.12688/f1000research.10065.1

**Published:** 2016-12-12

**Authors:** Berhane Ghebrehiwet

**Affiliations:** 1 The Departments of Medicine and Pathology, Stony Brook University School of Medicine, Health Sciences Center, New York, USA

**Keywords:** complement, innate immunity, cascade

## Abstract

The complement system, which consists of three independent but interacting pathways, constitutes a powerful arm of innate immunity. Its major function is to recognize and destroy pathogenic microorganisms as well as eliminate modified self-antigens. Although it is a fine-tuned system with innate capacity to discriminate self from non-self as well as danger from non-danger signals, an unwarranted activation can nonetheless occur and cause tissue destruction. To prevent such activation, specific regulators present both in plasma and on the cell surface tightly control it. Data accumulated over the past four decades have also shown that the complement system is capable of not only cross-talk with the activation cascades of plasma––i.e. blood coagulation, contact activation, and the kinin/kallikrein system––but also serving as a bridge between innate and adaptive immunity. It is for these reasons that the various activation steps of the complement system have been recently targeted for therapy to treat diseases in which the role of complement is beyond doubt. This trend will certainly continue for years to come, especially as novel concepts guiding the field into areas never contemplated before are continuing to be discovered.

## Introduction

At the last meeting of the “International Congress of Immunology”, which was held on the 21–26 August 2016 in Melbourne, Australia, there was a “spectacularly” heated but friendly debate between two groups––representing “innate immunity” on one side and “adaptive immunity” on the other––under a very creative title: “Adaptive Immunity is Innately Redundant”. Although the brilliant scientists in each group were theatrical at times and used colorfully funny language more befitting of a comedy circus than a scientific debate, the debate nonetheless brought to light the age-old question of whether adaptive immunity is a redundant bandage that only ensures a relapse does not occur after the initial damage has been properly taken care of by innate processes. What was equally intriguing––but not surprising––is that none of the discussants even mentioned the role of the complement system, which not only is one of the oldest members of innate immunity but also bridges both innate and adaptive immunity. Of course, in the end, not only are the functions of both the innate and the adaptive immune systems concatenated but also the crosstalk between the two systems ensures that foreign and modified self-antigens but not self-antigens are targeted for elimination.

Originally discovered as a system of innate immunity that “complements” the function of antibodies to kill or clear pathogenic microorganisms from the site of infection, the complement system has evolved to become much more than that. Accumulated data that span more than a century reveal that it is a highly complex and very tightly regulated effector system with the capacity to not only discriminate self from non-self but also ensure that even the non-self is innocuous with no “intent” to do harm. For example, the bacteria that are part of the commensal flora––i.e. non-self but with no “intent” to do harm––that are so abundant in our body, including in our digestive tract, are not normally targeted by the complement system, but infective microorganisms that enter our body to do harm are. In this manner, the complement system can target and eliminate pathogens and danger-associated molecular patterns by a variety of mechanisms including phagocytic and cytotoxic processes ^1^. An overview of the progress made in complement research is beyond the scope of this “opinion” article. Instead, this short review is meant only to highlight the present status and future direction of the field with particular emphasis on a few examples of complement-mediated diseases where targeted therapy has begun to make a difference.

## The complement system is a bridge between innate and adaptive immunity

**Figure 1.**
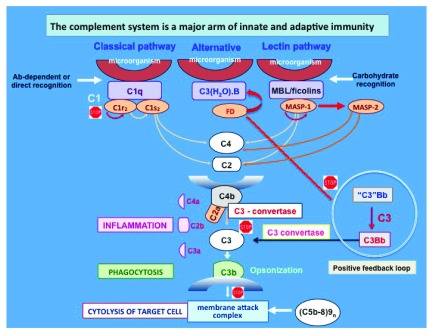
The three pathways of the complement system. The three independent pathways are known as the classical, alternative, and lectin pathways and were discovered in that order. Immune complexes activate the classical pathway, whereas the mannose-binding lectin (MBL) and alternative pathways are directly activated by mannose-rich or complex carbohydrate structures on pathogenic microorganisms, respectively. Regardless of how they are activated, the three pathways lead to the sequential activation of first C3 followed by C5, before the assembly of the membrane attack complex. The “stop” sign identifies critical steps that are or could be targeted for potential therapeutic interventions. Ab, antibody; FD, Factor D; MASP, mannose-associated serine protease.

The complement system consists of more than 50 plasma and cell surface proteins, which are organized to form three independent but interactive activation pathways. These are the classical, alternative, and lectin pathways, whose independent activation leads to the formation of the “killer” molecule known as the membrane attack complex (MAC) ( Figure 1). The MAC, which comprises C5b, C6, C7, C8, and C9n (C5b-9n, where n≥10), is responsible for the well-known complement-mediated 100 Å lesions seen on biological membranes by electron microscopy ^2– 8^. Because of its potential for tissue destruction, complement activation is strictly regulated by a plethora of enzyme inhibitors and regulators that act in concert at each critical step of the activation process ^1, 9– 12^. Endowed with the ability to recognize danger-associated molecular patterns, its major function is the recognition and elimination of not only pathogens but also modified self-antigens, such as those expressed on apoptotic self-waste ^1, 9– 12^.

Although the significance of complement in health and disease has been recognized almost since its discovery in the latter part of the 19 ^th^ century ^13– 16^, a number of critical discoveries made over the past several years, including the recent discovery of the mannose-binding lectin (MBL) pathway, have collectively underscored the importance of complement in both innate and adaptive immunity. Indeed, as an ancient member of innate immunity, it is designed to provide the requisite immunoprotection that is critical for survival in the face of infection. However, uncontrolled activation of complement due to either the presence of a high concentration of a potent activator (e.g. bacterial enzymes) or the deficiency of a regulator (e.g. decay accelerating factor [DAF or CD55] or protectin [CD59]) can lead to severe pathological conditions.

## Recent developments and therapeutic targets

Like most areas of immunology, the complement field has also benefitted immensely from recent advances in molecular biology, as well as from the development of sophisticated methods in biotechnology ^17^. Consequently, most of the proteins have been cloned, their chromosomal localization has been identified, their structure has been solved, and transgenic or knockout animal models have been generated ^18^. Therefore, we are now well past the era of diminishing returns and instead are better positioned to witness an accelerated growth in directions never imagined before. More importantly, we are discovering that the complement system is in fact a large network of cross-talking innate molecules––with built in checkpoints––that include the ever-expanding list of collectins, which are structurally and functionally similar to MBL and C1q ^6, 19^. With new discoveries also comes the realization that these molecules are involved in a wide range of intertwined pathological processes, each of which provides a target for the development of antibody-based or small-molecule-based therapy.

**Figure 2.**
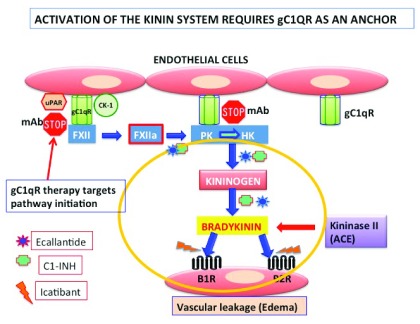
Activation of the kinin system. The kinin system is activated on the endothelial cell surface when Factor 12 (FXII) first binds to a tri-molecular receptor complex comprising gC1qR, urokinase-type plasminogen activator receptor (uPAR), and cytokeratin-1 (CK-1) and undergoes an autocatalytic conversion to generate FXIIa. FXIIa in turn converts prekallikrein (PK) to kallikrein, the enzyme that digests high-molecular-weight kininogen (HK) to generate bradykinin (BK). BK induces vascular permeability with the help of two receptors––bradykinin receptor 1 (B1R), which is inducible, and B2R, which is constitutively expressed on most cells. In the absence of C1-INH, uncontrolled generation of BK can cause vascular permeability resulting in angioedema (AE). The currently available therapeutic agents target specific checkpoints in the activation process, including a monoclonal antibody that prevents the binding of HK to gC1qR. Abbreviations: ACE, angiotensin-converting enzyme; mAB, monoclonal antibody.

Although the existence of cross-talk between the complement system and other cascades of plasma has been contemplated before, recent identification of novel molecules that play a major role in both the complement and the coagulation cascades have now rekindled this interest with more vigor and therapeutic purpose ^20– 22^. This is largely because C1-INH, the master regulator of the classical and lectin pathways of complement, is also the major regulator of contact activation, which leads to the generation of the powerful vasoactive peptide bradykinin ^20– 23^. Moreover, the unexpected discovery that the receptor for C1q, gC1qR––expressed on endothelial cells––is also one of the key proteins (the others being cytokeratin-1 [CK-1] and urokinase-type plasminogen activator receptor [uPAR]) that facilitates the assembly and activation of the kinin-generating pathway has also provided a novel platform for understanding the disease hereditary angioedema (HAE). This disease is caused by deficiency in C1-INH resulting in uncontrolled activation and generation of bradykinin ( Figure 2).

Despite the bias that has long existed––mainly by self-serving cellular immunologists who often doubted the relevance of complement as a powerful immunological system––some pharmaceutical companies, differing from this myopic view, have long understood the significance of complement in a broad range of pathological conditions to the extent that they have been engaged in devising ways of targeting the various steps of complement activation for the development of targeted therapy. By virtue of significance in the complement activation process, foremost among these targets have been C1, C3, and C5. Consequently, the first to be developed was Cinryze, which is an FDA-approved recombinant human C1 esterase inhibitor (C1-INH) that is now being successfully used to treat HAE ^24^. In addition to Cinryze, other therapeutic agents for the treatment of HAE have also been developed recently, including ecallantide (Kalbitor) or icatibant (Firazyr), which are based on their ability to block plasma kallikrein and B2R (bradykinin receptor 2), respectively ^23^.

Another FDA-approved drug is eculizumab (Soliris)––a humanized monoclonal antibody against C5––that was developed by Alexion to inhibit MAC-mediated cytolysis. It is therefore approved as a first-in-class complement inhibitor for the treatment of paroxysmal nocturnal hemoglobinuria (PNH), as well as for the treatment of atypical hemolytic uremic syndrome (aHUS) to reduce hemolysis ^25^. However, because of its effective blockade of the formation of the cytolytic MAC, the use of eculizumab will probably expand to treat diseases in which the cytolytic arm of complement plays a critical role. Another drug that is already in advanced pre-clinical trials before bedside intervention is compstatin ^26, 27^. Compstatin is a cyclic peptide that has now gone through several modifications and that inhibits complement activation by binding C3 and thereby interfering with convertase formation and C3 cleavage ^26^.

Encouraged by the success of the afore-mentioned drugs, there is now a growing list of small-molecule-based or monoclonal-antibody-based therapeutic modalities that are in the cocoon stages of development, and it won’t be long before they hit the market. Some of these, which are in either clinical or preclinical trials, include IFX-1 (InflaRx), which inhibits C5a and is geared for the treatment of septic shock; TNT-009 (True North Therapeutics), which targets C1s and is designed to treat cold agglutinin disease; and Bikaciomab (NovelMed Therapeutics), which inhibits Factor B and is used for the treatment of age-related macular degeneration ^28^. With continued advances in genetic engineering and stem cell biology, it will not be long before sophisticated techniques could be devised to reconstitute complement deficiencies (e.g. C1q, C2, or C4), which are almost invariably associated with diseases (e.g. systemic lupus erythematous and rheumatoid arthritis). The time is indeed approaching when cells making a deficient protein such as C1-INH or C1q are engineered outside the body and re-injected into the bloodstream to correct a given deficiency. The possibilities are therefore endless, and the future of complement can only be brighter.

## Evolving novel concepts and shifting paradigms

Although it is still in the early stages of conceptual development, one of the most exciting observations of the past few years is the discovery that key complement proteins are not only found inside the cell but also activated intracellularly in a manner similar to what occurs in plasma ^29, 30^. This unanticipated observation in turn led to the discovery that T cells contain endosomal and lysosomal pools of C3, which can then be processed into biologically active C3a and C3b by the T-cell-expressed protease cathepsin ^29^. Intracellular generation of biologically active complement fragments in turn may serve the cell for homeostatic survival, whereas translocation of these fragments may induce autocrine proinflammatory cytokine production ^29^. Although the existence of an intracellular pool of individual complement proteins is not a novel concept *per se*––since other cells have been shown to have functional pools of complement proteins ^31, 32^––it is the revelation of the existence of functional cross-talk between intracellular complement and the inflammasome that has ignited excitement in the field ^30^. The impact of this paradigm-shifting observation was already obvious at the recently held XXVI ^th^ International Complement Workshop in Kanazawa, Japan (4–8 September 2016), which dedicated a whole session to this discovery under the title of “Intracellular Complement”.

Another important area that is gaining interest––albeit a rekindled one––is the biological relevance of the local synthesis of complement proteins by cells. Although complement proteins are by and large synthesized in the liver, data accumulated over the past four decades have shown that individual complement proteins are synthesized by a wide range of cell types and are either secreted into the pericellular milieu or transiently expressed on the cell surface, where they regulate the function of the cells in an autocrine manner ^33, 34^. This is another area that is expected to reveal novel cellular players and unanticipated biological functions.

## Concluding remarks

The present overview is meant to guide the interested reader into the developing paradigm-shifting trends in complement research. Since complement’s discovery more than a century ago, research in this area has progressed slowly but steadily, with each phase revealing yet another unexpected function. However, each discovery was always met with the expected challenge by a few doubters, who tried to cast gloom. Although such discord always injected a healthy challenge, each discovery, by virtue of its conceptual and empirical strength, always stood the test of time. The recently discovered cross-talk between intracellular complement and the inflammasome is a case in point. It is an elegant display of conceptual brilliance and has already opened up a rich case to solve. In the words of George Bernard Shaw, “you see things; and you say “Why?” But I dream things that never were; and I say “Why not?”. It is the last part of this quote that we hope will be the driving force in the future if we are to make unexpected discoveries that will advance the requisite knowledge for the development of life-changing therapeutic options.
